# Supplementary material for: Low-Temperature Regulates the Cell Structure and Chlorophyll in Addition to Cellulose Metabolism of Postharvest Red Toona sinensis Buds across Different Seasons
Source: Int J Mol Sci. 2024 Jul 14;25(14):7719. doi: 10.3390/ijms25147719 (PMC11276666; doi:10.3390/ijms25147719)
Supplement: Supplementary file 1 [file ijms-25-07719-s001.zip › ijms-3038061-supplementary.pdf]

Table S1

Comparison of amino acids sequences in chlorophyll and cellulose metabolic pathway of *T. sinensis* with other species of woody plants

Toona sinensis			Woody plants			Consistency/%	E value
Gene name	Length/bp	Amino acids/aa	Reference species	Amino acid accession number	Amino acids/aa		
			Melia azedarach	KAJ4700698.1	429	89.50%	8e-131
TsALAD	750	249	*Turnera subulata*	KAJ4844740.1	251	89.69%	3e-115
			Citrus sinensis	KDO80610.1	327	86.55%	7e-132
			Melia azedarach	KAJ4701523.1	535	96.32%	0
TsHEMA	1308	435	*Durio zibethinus*	XP_022768534.1	551	92.18%	0
			Malania oleifera	XP_057973899.1	555	92.18%	0
			Pistacia vera	XP_031272347.1	371	89.46%	0
TsPBGD	1116	371	*Melia azedarach*	KAJ4710743.1	363	90.30%	0
			Citrus sinensis	KAH9771686.1	381	86.58%	0
			Melia azedarach	KAJ4726445.1	406	91.95%	0
TsUROD	1233	410	*Citrus sinensis*	XP_052294231.1	409	91.50%	0
			Pistacia vera	XP_031251373.1	409	88.29%	0
TsUROS	651	216	*Citrus sinensis*	KAH9758368.1	219	85.32%	2e-123

			Melia azedarach	KAJ4701801.1	299	90.09%	4e-121
TsCPOX	1185	394	*Citrus sinensis*	KAH9737438.1	395	89.11%	0
			Mangifera indica	XP_044462059.1	396	82.84%	0
TsCHLH	1683	560	*Sesbania bispinosa*	KAJ1429971.1	1383	95.00%	0
			Durio zibethinus	XP_022766664.1	1382	94.29%	0
			Morus notabilis	XP_010108425.1	418	94.48%	0
TsCHLI	981	326	*Ziziphus jujuba*	XP_015874235.3	421	94.17%	0
			Pistacia vera	XP_031275278.1	423	93.87%	0
TsCHLD	813	270	*Populus alba*	XP_034896575.1	765	87.27%	6e-133
			Populus trichocarpa	XP_006379057.3	766	86.89%	2e-132
			Melia azedarach	KAJ4728882.1	319	93.44%	0
TsChIM	963	320	*Citrus sinensis*	XP_006485173.2	322	88.82%	0
			Mangifera indica	XP_044502054.1	319	87.23%	0
			Melia azedarach	KAJ4722243.1	420	92.36%	0
TsDVR	1251	416	*Citrus sinensis*	XP_006489151.1	419	88.54%	0
			Hevea brasiliensis	XP_021656995.2	416	84.41%	0
TsGGR	1392	463	*Melia azedarach*	KAJ4712949.1	464	94.81%	0

TsCHLG	351	116	*Melia azedarach*	KAJ4704401.1	374	91.15%	1e-64
			Citrus sinensis	KAH9673049.1	337	86.73%	3e-61
TsCAO	1614	537	*Melia azedarach*	KAJ4700687.1	535	94.04%	0
			Citrus sinensis	KAH9757021.1	539	91.25%	0
			Mangifera indica	XP_044511410.1	535	89.94%	0
TsCesA1	1517	506	*Pistacia vera*	KAJ4707915.1	1083	95.05%	0
			Melia azedarach	XP_031286332.1	1085	95.84%	0
TsCesA2	3138	1046	*Juglans regia*	XP_018811669.1	1092	82.98%	0
			Carya illinoinensis	XP_042965459.1	1090	83.08%	0
TsCesA3	3294	1082	*Pistacia vera*	XP_031273752.1	1082	95.84%	0
			Mangifera indica	XP_044488972.1	1082	95.74%	0
TsCesA4	2211	737	*Alnus glutinosa*	XP_062148107.1	1048	86.97%	0
			Mangifera indica	XP_044493658.1	1051	89.99%	0
TsCesA5	1470	489	*Prunus avium*	XP_021813286.1	1056	86.57%	0
			Macadamia integrifolia	XP_042507470.1	1091	88.07%	0
TsCesA6	1140	379	*Populus euphratica*	XP_011036830.1	1110	93.05%	0
			Prunus dulcis	XP_034197686.1	837	91.82%	0

			Prunus avium	XP_021813287.1	836	91.56%	0
			Citrus sinensis	KAH9654321.1	1070	94.95%	0
TsCesA7	1545	515	*Mangifera indica*	XP_044472541.1	1042	91.30%	0
			Tripterygium wilfordii	XP_038709999.1	1041	89.15%	0
			Citrus sinensis	KAH9797391.1	979	93.89%	0
TsCesA8	2421	806	*Mangifera indica*	XP_044461676.1	977	90.72%	0
			Pistacia vera	XP_031253638.1	1375	90.76%	0
TsCslB4	861	286	*Melia azedarach*	KAJ4730065.1	752	80.31%	2e-138
TsCslD3	3369	1122	*Citrus sinensis*	XP_006471487.2	1115	82.59%	0
			Pistacia vera	XP_031266877.1	1219	81.30%	0
			Pistacia vera	XP_031288134.1	1170	88.87%	0
TsCslD5	1880	627	*Citrus x clementina*	XP_006444438.1	1165	88.85%	0
			Citrus sinensis	KAH9730655.1	655	88.50%	0
TsCslE6	1278	425	*Pistacia vera*	XP_031270370.1	739	75.23%	0
TsCslG2	1161	386	*Citrus sinensis*	KAH9682165.1	774	78.47%	6e-163
TsCslG3	1317	438	*Melia azedarach*	KAJ4711858.1	738	83.11%	0

Table S2

Primers for qPCR related to chlorophyll and cellulose synthesis

Gene	Forward primer (5'-3')	Reverse primer (3'-5')	Substance
TsALAD	GCCTGGATGTTATAGACTTGGAT	GCACTAGACCGTTCTCATTGTAT	Chlorophyll
TsHEMA	ATTCATTGGAGACTGTTCCTACC	TTATTCACGATACCACGGCTAAG	Chlorophyll
TsPBGD	TATCAGAATTGGCACTAGAGGAAG	TTCATAGAATGTACGGCGATGTC	Chlorophyll
TsUROD	GAAGGTATATGGCTGCTTACAGA	GAACGAATAGGTGACTGAATAATGG	Chlorophyll
TsUROS	CCAATGTTAGGGTAGGAGTTGTG	CAGAAGCAGGATACAAGACAGTG	Chlorophyll
TsCPOX	TATATCTTCGAGGAGGATGTTAAGC	GTATGTAAGCAGGAATCACGGAAT	Chlorophyll
TsCHLH	CTGGTGTAGGAATGACTGAGAAT	CTGTGGTAGTATCAGCAATGTATG	Chlorophyll
TsCHLI	GATGCCGAACTCAGAGTAAAGAT	GTCTCCTCTCAATCCATCAACAT	Chlorophyll
TsCHLD	TCATTCTTCCTCGTTCGATTGT	GGTATCTGTTCCTGTTGTTGTTC	Chlorophyll
TsChlM	GTTGAAAGGAGTAACCGTTTGTG	CTAAGCAGACTACCGTGTCATAC	Chlorophyll
TsDVR	GGAAGGAACCAAAGTTCTTGAAAG	ACTATAATCGCCAGTCTCAGGAT	Chlorophyll
TsGGR	GGTGATTATGAATACGCCATTGC	CTTGTTGCTAGTTGGAACTTCTTG	Chlorophyll
TsCHLG	CACAAGGAGATTAACGGTGAGAG	GCAGACGAATCTTCCATTTGTTAG	Chlorophyll
TsCAO	TGCCACTGCCTTAGATAAACTAG	TGTAAGATTGAACTGGACCTGAG	Chlorophyll
TsCesA1	TCTATACTTGCTGTGGATTACCC	CTCTAGGCTCTATATTGTGCTTCT	Cellulose
TsCesA2	GAGTGGAGTAAGCATCGAAGAAT	GTCGGAGGAATTAGAAGAGTTGT	Cellulose
TsCesA3	TCCAATCACTGCTATTCCTCTTC	CCACCATTCATCAATTCCAACAC	Cellulose
TsCesA4	CAGGAAGCGTTAATGGTAAGGAT	CGGAGGATAATGACTATGCGATAT	Cellulose
TsCesA5	GTGGTTCTGACTCCGATTTACAT	AGCAACACTTCCATAGCCATAAG	Cellulose
TsCesA6	GTTGGTATTGATGAATGGTGGAG	ACTATAAGCAGTGTTGTTGGAGG	Cellulose
TsCesA7	GAAGGAGATGACGATGAAGAAGA	GCTATAACTGGTGGGAATTGAGA	Cellulose
TsCesA8	TTATGGTCTATGGCTCACTTCTG	CTTTCAATGGGTCAACTGTACTC	Cellulose
TsCslB4	GCCGATGATGAGTTACTAAAGAAG	GAGCCATATAACCAGCCAACATT	Cellulose
TsCslD3	CATTCACCTCCATATTCCTCATTG	CTCATTCCTCCACCATTCTTCTA	Cellulose
TsCslD5	AGGATTCAATTCAGTGACGAGAG	CATTATCTCCATCGCTAACCTCT	Cellulose
TsCslE6	TGTAAGACAGACTGGAAGAGAAC	CCTCCGCATTGTATCAATAATCC	Cellulose

TsCslG2	CAAAGTTCTGCCCTCCTATTTATG	GTGATGATAACCTCCTCCAAGTG	Cellulose
TsCslG3	CTAACTATGTCGGAACTGGAACC	CTCTGCCAACGAACCATATCTAA	Cellulose
TsActin	GGTCAGAAGGATGCCTATGTTG	GGGATTTAGAGGAGCCTCAGTT	Actin
